# Cosmetic outcome of skin adhesives versus transcutaneous sutures in laparoscopic port-site wounds: a prospective randomized controlled trial

**DOI:** 10.1007/s00464-015-4474-5

**Published:** 2015-10-01

**Authors:** Olaf Buchweitz, Christian Frye, Claus Peter Moeller, Wolfgang Nugent, Eckart Krueger, Andreas Nugent, Peter Biel, Sven Juergens

**Affiliations:** Tagesklinik Altonaer Strasse, Altonaer Str. 57-61, 20357 Hamburg, Germany; Wound Market Consulting, Heyford, Oxfordshire UK; Department of Gynecology and Obstetrics, University Hospital, Münster, Germany; Department of Gynecology and Obstetrics, University Hospital, Cologne, Germany

**Keywords:** Cosmetic outcome, Laparoscopy, Octyl-cyanoacrylate, Port-site incisions, Skin adhesive

## Abstract

**Background:**

In an elective laparoscopic surgery, the cosmetic outcome becomes increasingly important. We conducted a study to evaluate the cosmetic outcome 3 months after a laparoscopic procedure and compared skin adhesive (SA) versus transcutaneous suture (TS).

**Methods:**

A randomized, controlled, prospective study was conducted at a single study centre in Hamburg, Germany. Seventy-seven patients undergoing laparoscopic surgery with two lower abdominal port sites met the study requirements. It was decided randomly which port site would be closed with SA. The opposite site was closed with TS. Wounds were assessed after 7–12 days and after 3 months. Cosmetic outcome was measured by a visual analogue scale (VAS) completed by the patient, by the Hollander wound evaluation scale (HWES) and by the judgement of blinded investigators.

**Results:**

Seventy-seven subjects were randomized. Complete data from the 3-month follow-up visit were available from 56 patients (72.7 %). The VAS scale ranged from 0 to 100 mm with “0” representing the best possible cosmetic outcome. Median satisfaction was 2 mm in the TS group and 3 mm in the SA group. The mean was high in both groups 4.6 (*s* = 13.1) versus 3.8 mm (*s* = 4.6). The outcome was neither clinically nor statistically significant. Cosmetic outcome was assessed by an investigator, and the HWES showed no difference. In regard to complications, no difference was found between SA and TS, either.

**Conclusions:**

In conclusion this study demonstrated that closure of laparoscopic port-site wounds leads to equivalent outcomes whether SAs or TSs are used. Complications are rare in both methods. Thus, SAs seem to be a valid alternative to sutures in laparoscopic surgery.

Registration site: www.clinicaltrials.gov.

Registration number: NCT02179723.

**Electronic supplementary material:**

The online version of this article (doi:10.1007/s00464-015-4474-5) contains supplementary material, which is available to authorized users.

Diagnostic and operative laparoscopies are among the most common procedures in gynaecology and abdominal surgery departments. The port-site wounds are small, are usually under low tension and have a low rate of impaired wound healing/complications.

There is no “gold standard” for the method of wound closure in laparoscopic wounds. A variety of procedures is available such as transcutaneous suture, subcuticular suture, adhesive paper tape, skin staples and more recently skin adhesives. Surgeons appear to choose a technique based on their individual experience and preference. Other criteria for the choice of the wound-closing technique include patients’ satisfaction, costs or time needed for wound closure.

In an elective laparoscopic surgery, the cosmetic outcome becomes increasingly important. This need is reflected by two different strategies: on the one hand the development of single-incision laparoscopic surgery and on the other hand the endeavours of the industry to design smaller and smaller trocars for multiport laparoscopy. However, even though single-incision laparoscopic surgery has been projected to have better cosmetic outcomes compared with conventional laparoscopic procedures, there are no convincing data to support this [1–3]. Surprisingly, there are also very few data concerning cosmetic outcome with the different wound-closing methods in multiport laparoscopic surgery.

A recent Cochrane review [4] identified only one randomized clinical trial suitable for the meta-analyses giving data on cosmetic results 3 months after a laparoscopic procedures [9].

When a new skin adhesive (Leukosan Adhesive^®^, BSN medical GmbH) recently became available in Germany, we conducted a study to compare skin adhesive versus transcutaneous sutures in laparoscopic port-site incisions. Transcutaneous sutures as a comparator were chosen as a previous study performed in our clinic had shown that transcutaneous sutures in laparoscopic surgery seemed to be the most suitable technique for the closure of laparoscopic port-site incisions compared with subcuticular sutures and adhesive tapes [5].

The present study is the first randomized clinical trial comparing skin adhesive versus transcutaneous suture which was specifically designed to evaluate the cosmetic outcome as the primary endpoint at 3 months after the laparoscopic procedure.

## Materials and methods

### Study design, setting and population

The study was designed as a randomized, controlled, prospective and mono-centred study. Ethics approval was obtained from the ethics board responsible: Ethikkommission der Ärztekammer Hamburg. Institutional approval was granted by Tagesklinik Altonaer Strasse. From March 2012 to April 2013, all patients referred for laparoscopic surgery at the gynaecology day clinic (Tagesklinik Altonaer Strasse) in Hamburg, Germany, were asked to participate in the study. Inclusion criteria were: women older than 18 years and not older than 60 years, planned laparoscopy with two mirrored trocar wounds, willingness to come for wound assessment after 7–12 days and after 3 months and informed consent. Exclusion criteria included laparoscopy with duration of more than 2 h, intraoperative need to enlarge trocar wounds thus leading to different wound sizes and diabetes mellitus.

All incisions were made identically in the lower abdomen to place a 5-mm trocar. The closure technique of the two lower abdominal wounds was randomized to skin adhesive (Leukosan^®^Adhesive, BSN Hamburg, Germany) or transcutaneous suture (Premilene^®^ DSMP 24, 3/8 needle, thread size 3/0, B. Braun, Melsungen, Germany).

Immediately before closing the wound, a final check of the inclusion and exclusion criteria was made. Randomization was carried out by means of a sealed envelope containing the location of the port to be closed with the skin adhesive. The randomization envelopes were provided by an external centre (Wound Market Consulting). On the basis of the subject identification number, the numbered envelopes were opened by the investigator or an assigned person, in most cases the anaesthesiologist. The opening of the randomization envelope had to be documented by the signature of the investigator.

The umbilical trocar incision was always closed with suture. All sites were covered with a self-adhesive opaque plaster. Patients were instructed to remove the plaster after 72 h.

Three different tools were used to measure the cosmetic outcome. After 3 months, we assessed the patient’s satisfaction with the cosmetic result using a visual analogue scale (VAS). At the same time, the Hollander wound assessment scale (HWES) was used by a blinded investigator [6]. A forced choice question for the blinded investigator (“Which site looks better?”) was documented at 7–12 days as well as at 3 months after surgery.

Other secondary endpoints were the incidences of complications and of pain. The latter was measured with a VAS by the patient. All patients were asked to attend an assessment 7–12 days and 3 months (10–14 weeks) after surgery at the study site. At the first assessment, the stitches were removed.

### Sample size calculation

The primary scope of the study was to demonstrate equivalence between skin adhesive and transcutaneous sutures. Sample size was calculated in order to demonstrate that the mean treatment difference in primary endpoint (cosmetic outcome measured in VAS scale 0–100 mm) was contained inside the interval [−10 mm; 10 mm], which was considered to be a clinically irrelevant difference. This was done by performing the two one-sided *t* tests approach on the paired treatment differences. For these tests, type I error of 0.05 was chosen, i.e. the level of significance *α* = 5 %.

In the similar study by Chen et al. [7], the total HWES score was around 5.5 and showed a maximum standard deviation of about 0.13 (i.e. the variation coefficient not higher than 2.5 %). Considering the equivalence range 10 mm and standard deviation not higher than 15 mm, a sample size of 40 subjects is sufficient to reject the null hypothesis given the significance level of 0.05 and power of 0.8. Taking into account the relatively high expected dropout of around 30–40 %, up to 60–70 patients were planned to be enrolled.

### Statistical analysis

The primary endpoint was the patient’s satisfaction with the cosmetic outcome of the healing of the port sites at 3 months post-operative, as measured by a VAS. To demonstrate the equivalent efficacy of both treatments, the VAS evaluations were further analysed by testing the hypothesis H0. The mean treatment difference is not within the equivalence range [−10 mm; 10 mm]. As each subject had two sites with each of them treated by a different product (skin adhesive vs. transcutaneous suture), the statistical comparison of treatment groups for cosmetic outcome had to be analysed as paired differences. We planned to use the Student’s *t* test to calculate 90 % confidence interval for mean. However, as the data were not normally distributed, the nonparametric rank statistics were used to estimate 90 % confidence intervals for the median:

$${\text{Median}}\left( {\Delta {\text{VAS}}} \right) = {\text{median}}\left[ {{\text{VAS}}\left( {\text{Transcutaneous}} \right){-}{\text{VAS}}\left( {\text{Leukosan}} \right)} \right]$$

Chi-square test was used to compare categorical data and Student’s *t test* for metric variables except for the primary outcome (see above). *P* values smaller than 0.05 were considered significant.

The statistical analysis was carried out using the statistical package SAS, version 9.3.

## Results

**Fig. 1:**
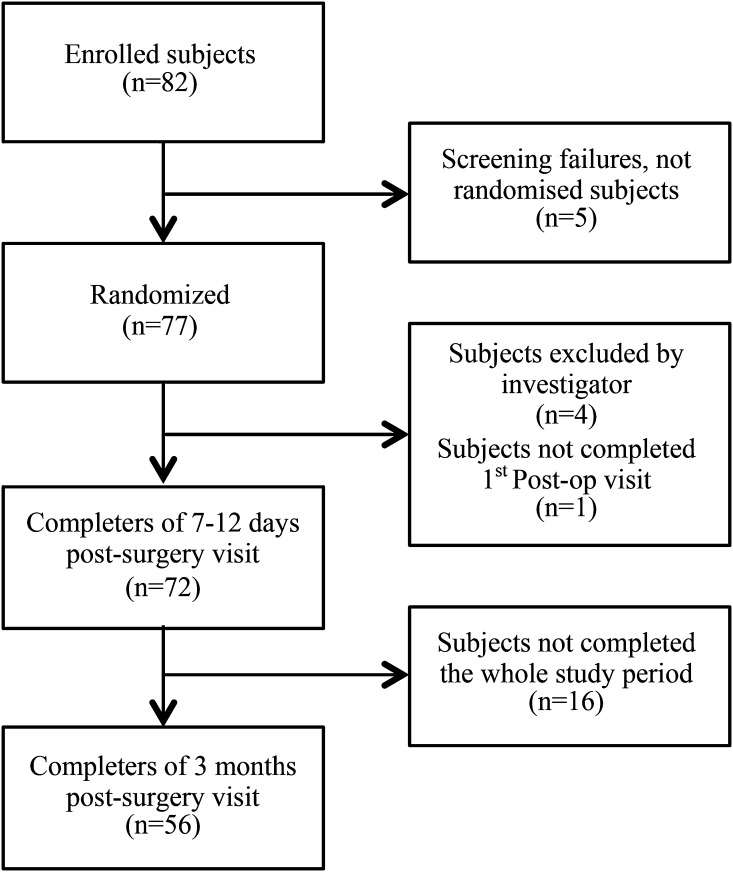
Study population

A total number of 82 patients were asked to participate in the study. Seventy-seven subjects were randomized. Complete data from the 3-month follow-up visit were available from 56 patients (see Fig. 1). The mean age of the women was 35.6 years in all patients randomized, and infertility was the main reason for laparoscopic surgery. The mean length of the wound was 0.62 cm. Table 1 shows the characteristics of all patients randomized as well as of all subjects who completed the 3-month visit (*n* = 56).

**Table 1:** Characteristics of subjects randomized and subjects with complete data at 3-month follow-up

	Patients randomized (*N* = 77)	Completers of 10–14 weeks post-surgery (PPS) (*N* = 56)
Variable		
Age, years (SD)	35.60 (8.81)	35.77 (8.72)
Height, cm (SD)	167.68 (6.39)	166.59 (6.25)
Weight, kg (SD)	65.39 (12.77)	63.70 (11.59)
BMI, kg/m^2^ (SD)	23.20 (3.92)	22.89 (3.55)
Smoking (never)	72.7 %	71.4 %
Indication for laparoscopy (%)		
Infertility	46.8	44.6
Endometriosis	50.6	51.8
Adhesion	19.5	19.6
Ovarian cysts	31.2	37.5
Other	15.6	10.7
Trocar wound characteristics		
Length, cm of incision (SD)	0.62 (0.27)	0.65 (0.29)
Location of wound—lower abdomen	100.0 %	98.2 %
Location of wound—supra pubic	–	1 (1.8 %)

Mean (SD) for metric variables, percentage for categorical variables

The primary endpoint was defined as the satisfaction of the patients with the cosmetic outcome in the two lower abdominal laparoscopic port-site wounds after 3 months. It was assessed by the patients with a VAS 3 months after laparoscopy. The VAS scale ranged from 0 to 100 mm with “0” representing the best possible cosmetic outcome and “100 mm” representing the worst possible cosmetic outcome. The mean satisfaction with the port-site wound was slightly higher in the skin adhesive wounds 3.8 mm (*s* = 4.6) compared with the wounds closed with transcutaneous sutures (mean: 4.6 mm; *s* = 13.1). The mean of paired differences found was 0.84 mm (s = 13.35). However, as the confidence interval for the paired differences includes “0”, the slight difference in satisfaction with the cosmetic outcome was not statistically significant. Median satisfaction was 2 mm (range 0–92 mm; 95 % CI 0.0; 3.0) in the transcutaneous suture group and 3 mm (range 0–21 mm; 95 % CI 1.0; 4.0) in the skin adhesive group.

Cosmetic outcome was also evaluated with a forced choice question by a blinded investigator. At 7–12 days after surgery, wounds were judged to look a little better in the skin adhesive than in the transcutaneous suture group (57 vs. 41 %). At assessment after 3 months, there was no difference whatsoever (see Table 2).

**Table 2:** Cosmetic outcome

	Transcutaneous suture	Skin adhesive	Paired differences Transcutaneous sutures—skin adhesive
Satisfaction with cosmetic outcome (VAS) 10–12 weeks post-surgery as judged by subject^a^			
Mean (SD)	4.64 (13.10)	3.80 (4.60)	0.84 (13.35)
Median (range)	2 (0; 92)	3 (0; 21)	0 (−17; 89)
95 % CI for median	(0.0, 3.0)	(1.0, 4.0)	(−2.0, 0.0)
Cosmetic result judged by investigator (which treatment looks better) (%)			
7–12 days post-surgery (*n* = 55)	41.8	58.2	
10–12 weeks post-surgery (*n* = 56)	50	50	
Evaluation by HWES 10–12 weeks post-surgery			
HWES scale 0	56 (100.0 %)	55 (98.2 %)	
HWES scale 1	–	1 (1.8 %)	

^a^Subjective evaluation of cosmetic effect was done using VAS scale 0–100 mm (0 best possible outcome; 100 worst possible outcome)

With regard to complications (Table 3), no difference was found between skin adhesives and transcutaneous sutures. After 7–12 days, all but one wound were closed in the transcutaneous suture group and all but two in the skin adhesive group. Five incisions closed with transcutaneous suture showed reddening versus none in the wounds closed with skin adhesives. Three months after surgery, all wounds were closed in both groups. There was no wound with dehiscence, secretion or redness in either group. The mean pain level assessed by VAS was 0.88 mm in incisions closed with transcutaneous suture versus 0.96 mm in incisions closed with skin adhesives.

**Table 3:** Complications of wound healing and self-reported pain 7–12 days and 10–14 weeks post-surgery

	Transcutaneous suture	Skin adhesive
Post-operative (7–12 days)		
Suture closed	55 (98.2 %)	54 (96.4 %)
Dehiscence	–	1 (1.8 %)
Secreting	0	0
Redness	5 (8.9 %)	–
Pain (VAS mm) *N* = 55		
Mean (SD)	8.87 (15.09)	5.62 (8.07)
Min/median/max	0/3/69	0/2/33
Post-operative (10–14 weeks)		
Suture closed	56 (100.0 %)	56 (100.0 %)
Dehiscence	–	–
Secreting	–	–
Redness	–	–
Pain (VAS mm) *N* = 56		
Mean (SD)	0.88 (1.31)	0.96 (1.21)
Min/median/max	0/0/5	0/0/4

Mean (SD) for metric variables, percentage for categorical variables

## Discussion

Cosmetic satisfaction is an important outcome for patients after surgery, and this is most likely especially true for young women. Generally, wound modelling at 3 months is expected to provide an indication of eventual scar evolution even if complete remodelling may take up to 24 months. This is supported by the results of Quinn et al. [8], who reported that the cosmetic outcome after 3 months is strongly predictive of the cosmetic appearance after 1 year.

A Cochrane Review from 2010 investigated tissue adhesives for closure of surgical incisions. The meta-analyses also included the cosmetic outcome. However, the authors excluded all studies with data taken at a time point of less than 3 months following surgery.

The present study is the first randomized clinical trial comparing skin adhesive versus transcutaneous suture which was specifically designed to evaluate the cosmetic outcome as the primary endpoint at 3 months after the laparoscopic procedure.

All previous studies comparing tissue adhesive with sutures in laparoscopic port-sites reported primarily on wound-closing time [9–13] or early complications [7] and only tracked cosmetic results as a secondary outcome. None of them reported statistically significant differences in cosmetic outcome, but only two of these assessed the cosmetic result after 3 months [9, 10].

Our findings correspond well to the only two other studies that tracked cosmetic outcomes of laparoscopy for a period of 3 months post-surgery: the studies by Maartense et al. [9] and Dowson et al. [10]. Maartense et al. employed a VAS to measure the cosmetic results, whereas Dowson et al. applied the HWES. However, both papers reported no difference in the cosmetic outcomes. One major difference of the present study compared to the studies by Maartense and Dowson is that in our study every patient served as their own control through the use of highly standardized mirrored port sites that were randomly assigned to one of the two closing methods. We believe that this is a very strong design. Known and unknown potential confounders are eliminated when each patient serves as their own control. Important sources of bias such as allocation bias, selection bias or loss of follow-up bias are thus even more unlikely than in a regular RCT.

The satisfaction with the cosmetic result in our study was extremely high. Both skin adhesives and sutures were within a 5 % range of the “best possible result”. Comparing the VAS values of the patients’ satisfaction with skin adhesives versus sutures, Maartense reported a mean of 76 vs. 78 mm after 3 month (with 100 mm being the best possible outcome) [9]. We believe that the excellent cosmetic results in our study are mainly due to the following reasons: firstly, we used transcutaneous sutures which had shown better cosmetic results than subcuticular sutures in a previous study [5], and secondly, we compared only the “identical” lower abdominal port sites with each patient as their own control. For methodological reasons, the slightly larger umbilical port was not part of the cosmetic assessment in this study.

The Cochrane Review from 2010 cited above investigated tissue adhesives for closure of surgical incisions. The primary outcome of the meta-analyses was the proportion of wounds breaking down (wound dehiscence). The review concluded that sutures were significantly better than tissue adhesives for minimizing dehiscence [4].

However, this report did not focus solely on laparoscopic incisions but included a variety of other surgical wounds. The Cochrane review only includes one study with regards to dehiscence comparing sutures and skin adhesives in laparoscopic port-site wounds. This was the study by Dowson et al. [10] published in 2006. Dowson reported no significant differences in wound complications or in cosmesis at either 4–6 weeks or 3 months. However, there were four cases of dehiscence in wounds closed with skin adhesives versus zero in the suture group 24–48 h after surgery. After the deadline of the Cochrane Report literature search in Nov. 2009, two additional randomized studies specifically investigating port-site closure with skin adhesive versus sutures were published [7, 13]. Both of them as well as our trial showed no increased risk of dehiscence using skin adhesive for the port closure. We believe, therefore, that the use of skin adhesive for laparoscopic procedures is safe and not associated with more cases of dehiscence than wounds closed with sutures.

There is good evidence that skin adhesives save operating time compared with sutures in closing port-site incisions [9–13] and other surgical wounds [14–18].We did not measure operating time, but when asked, the surgeons were of the firm opinion that closing the wounds just with the skin adhesive was at least as fast as closing the wounds with transcutaneous sutures. Another important advantage of the skin adhesive is the fact that there is no need to remove the threads after surgery. While removing the thread is quite easy at the trocar wounds located at the lower body, it is rather disturbing at the umbilical port site.

Our study supports the fact that skin adhesives and sutures have a comparable low rate of complications in laparoscopic procedures. Furthermore, our study gives strong evidence that wound closure of laparoscopic port-site wounds by either skin adhesives or transcutaneous sutures leads to an equivalent cosmetic outcome. Skin adhesives seem thus to be a valid alternative to sutures in laparoscopic surgery.

## Electronic supplementary material

Below is the link to the electronic supplementary material.

Supplementary material 1 (DOCX 86 kb)
